# Cancer disparities: an extensive review

**DOI:** 10.3332/ecancer.2026.2134

**Published:** 2026-05-27

**Authors:** Chinaecherem P Okafor, Chekwube K Nwufo, Victor Akachukwu Ibiam

**Affiliations:** 1 Department of Biochemistry, City University of New York, The City College of New York, 160 Convent Avenue, New York, NY 11031, USA; 2 Department of Biochemistry, Federal University of Technology, Owerri, Imo PMB 1526, Nigeria; 3 Biochemistry Division, Department of Chemistry, Purdue University, West Lafayette, IN 47907, USA; 4 Department of Community Engagement, Divine Purpose Community Services LLC, Baltimore, MD 21209, USA

**Keywords:** cancer disparities, race, breast, prostate, colon, cervical, lung

## Abstract

Cancer disparities are persistently a significant public health burden worldwide and disproportionately impact the underserved populations. To effectively combat cancer disparities, a clear understanding of their causes and patterns is essential. Currently, government data and peer-reviewed research journals display a wide range of data to describe the extent of different cancer disparities. However, there are still gaps in understanding the scope and patterns of these disparities and in adopting practical approaches to eliminate them adequately. Accordingly, we examine essential topics that are crucial to a coherent comprehension of the causes and patterns of major cancer disparities. The complex relationship between the different determinants of health inequalities creates unique patterns of disparities for different cancer forms and populations. Addressing these disparities will require diverse research approaches with rigorous frameworks from multiple disciplines. This review takes an in-depth study into cancer disparity patterns to inform effective intervention strategies for underserved populations. We present a comprehensive analysis of disparities in breast, colorectal, lung, prostate and cervical cancers. We analysed the interplay between genetic, socioeconomic, geographic, behavioural and environmental factors that shape cancer outcomes. Drawing inferences from recent studies on cancer epidemiology and intervention programs, this review highlights how underserved populations are disproportionately affected due to systemic barriers to early diagnosis, effective treatment, screening access and health literacy. Disparity patterns in incidence, mortality and survival rates were comprehensively described using recent studies, while also evaluating community-based interventions and policy efforts intended to address these disparities and mitigate their impacts.

## Introduction

Despite global health campaigns and research efforts, cancer remains the Achilles’ heel of the health system. As a result, cancer has emerged as a significant global public health concern in recent years, ranking among the leading causes of death in many countries. In the United States, for example, cancer is a significant cause of mortality, resulting in 613,362 deaths in 2023 and second only to cardiovascular conditions [1].

With the ever-growing size of the world population and the increasing aging of the current generation, the cancer burden has rapidly increased in recent decades [2]. Cancer burden varies across different populations and is usually influenced by genetics, race, gender, age, ethnicity and socioeconomic factors [3, 4]. Consequently, notable disparities in cancer prevalence, incidence and mortality rates have been documented in various research materials. These disparities are notably attributed to factors such as those listed earlier. Cancer disparity is evident in the United States and other parts of the world. The American Cancer Society reports that the cancer burden varies across different cities in the country and individual States [5]. For instance, Southern states and Appalachia have the highest incidence of early-onset colorectal cancer (CRC), with Kentucky having the highest rate (14.3 cases per 100,000 people aged 20 to 49) between 1995 and 2015. While southern states show a higher prevalence of early-onset CRC than other regions, western states demonstrate the lowest rates [6]. Similarly, cancer disparities across different races and ethnicities are well documented in the United States. Zahm and Fraumeni [4] reported that Blacks are more predisposed to most types of cancers, with incidence rates about three times higher for esophageal cancer, two-fold higher for liver, cervical, multiple myeloma and stomach cancers, and about 1.5-fold higher for larynx, pancreas and prostate cancers, while lung cancer is prevalent among whites. These statistics suggest that cancer disparity is a major public health concern in the United States.

To effectively address the health burden placed on underserved groups due to cancer disparities, it is crucial to understand the etiology of these disparities. Over time, descriptive studies uncovering patterns and incidence of diseases among populations have helped provide insights into the etiology of cancer disparities. These studies are instrumental in tracking disparities and may be an essential pointer to environmental and health hazards. Just as in many other diseases, descriptive studies on cancer disparities have been crucial in uncovering the pattern of cancer prevalence, progression and mortality. In addition, studying cancer disparities has been used to evaluate the impact of various public campaigns against cancer, such as cancer prevention, diagnosis and treatment progression, and to predict future trends that may help set priorities in cancer research. Given the importance of studying cancer disparities, it is expedient that more information on the pattern and disposition of cancer disparities be provided to guide researchers and health agencies in combating the health burden caused by cancer disparity.

While multiple reviews have addressed cancer disparities, most tend to focus narrowly on individual factors, lacking an integrated, multifaceted analysis that encompasses the complex interactions of social, biological and environmental determinants. In this study, we present a narrative review that broadens the analysis of the already rich library of information on cancer disparities and provides more insight into the pattern of cancer disparities across various vulnerable populations. Our review provides an interdisciplinary synthesis of recent data and practical case-to-case comparisons that uniquely highlight how the intersection of biological and social determinants shape cancer outcomes across populations. We also analysed community-based interventions and policy implications, hence bridging the gap between descriptive epidemiology and practical strategies in mitigating cancer disparities. We also provide insight into future epidemiologic research that may increase the understanding of the impacts of race, ethnicity and gender on cancer risk.

### Literature search strategy

Our search strategy involves sourcing recent data from major peer-reviewed journals, government data and epidemiological databases to gather recent and relevant studies on cancer disparities. Data from peer-reviewed journals were gathered through the prompting of Google Scholar (a multidisciplinary search engine), Pubmed Central (a specialized search engine) and Google Search. Major prompts include ‘cancer’, ‘cancer disparities’, ‘liver cancer’, ‘lung cancer’, ‘CRC’, ‘prostate cancer’, ‘cancer advocacy’ and ‘cancer disparity intervention’. Our primary government data sources are the United States Cancer Database, including the United States Cancer Statistics, the Surveillance, Epidemiology and End Results Program and the National Cancer Database. Our inferences are based on case-by-case comparisons across major cancer types.

### Historical context of cancer disparities

Historically, cancer disparities are shaped by a complex interplay of social, economic, environmental and biological factors [7]. For instance, research in the US has indicated that those of lower socioeconomic position and members of racial/ethnic minority groups had a greater incidence of cancer, underscoring the necessity of focusing interventions on these groups to alleviate cancer disparities. Cancer has dramatically impacted various communities in a variety of contexts as the world’s top cause of morbidity and mortality [8]. To successfully address these disparities, it is imperative to comprehend the historical backdrop.

In the early 1900s, cancer diagnosis and treatment were rudimentary, and disparities were less documented, mainly due to a lack of comprehensive data. Cancer care was primarily accessible to affluent populations, leading to a significant underreporting of cases among marginalized communities [9]. After the Second World War, advances in medical technologies and the establishment of national cancer registries in the mid-20th century led to better documentation of cancer cases. However, the benefits of these advancements are not equally distributed. Blacks, rural populations and those with lower socioeconomic status (SES) often faced barriers in accessing cancer care, including limited availability of screening and treatment facilities [9].

Following the civil rights movement and increased awareness of healthcare inequities between the late 20th and early 21st century, efforts to address cancer disparities began to take shape. However, despite progress, significant disparities persisted. The introduction of targeted therapies and advanced screening techniques in the late 20th century improved survival rates, but access to these innovations remained uneven, exacerbating existing disparities. DeLancey *et al* [10] reported a marked decrease in the overall Black-White disparity in cancer mortality between 1990 and 2004. The decrease was primarily attributed to decreases in tobacco-related cancer mortality and highlights the success of campaigns against health inequalities. However, racial disparities increased for cancers whose outcomes can be affected by screening and treatment timings. Therefore, there is a need to improve early detection and treatment across all population segments to eliminate these disparities effectively.

## Major types of cancer affected by disparity

Cancer disparities are not uniform across all cancer types. Certain cancers exhibit more pronounced disparities due to risk factors, access to care and biological differences. Recent studies have shown that socioeconomic inequalities most strongly contribute to disparities in cervical, colorectal, lung and liver cancers [11]; genetic factors are more likely to affect breast, prostate, ovarian and cervical cancers, while racial disparities are well documented for prostate (Blacks) and lung (Asians) cancers [12]. While it is difficult to effectively capture the detailed disparity pattern of all cancers in a single review, we provide a deep insight into the disparity of five major cancer types that contribute to the significant proportion of global cancer incidence, mortality and observed disparities across different population types. These cancer types include breast, colorectal, lung, prostate and cervical cancer. They represent both male and female cancers, span diverse biological systems, and are heavily influenced by social, economic, genetic and geographic factors. Additionally, public health efforts and intervention programs often prioritize these cancers, making them broad representatives of cancer disparities and key indicators for evaluating the success of disparity reduction strategies.

**Breast cancer**: As the second most common form of cancer worldwide, breast cancer poses a significant threat to global health. With an estimated 313,510 cases in 2024 [7], it is one of the most frequently diagnosed cancers in the United States. Globally, it was the most diagnosed cancer in 157 countries and the primary cause of cancer-related fatalities in 112 countries in 2022 [13]. The worldwide age-standardized incidence rate for females is 48 per 100,000, varying from around 30 per 100,000 in sub-Saharan Africa to over 70 per 100,000 in Western Europe and North America [14]. Although the highest relative incidence of breast cancer is found in developed regions, more than 50% of breast cancer incidence are recorded outside of the high-income countries (HICs) because of the overwhelmingly high population of less developed countries. This scenario results in a significant disease burden, with about two-thirds of annual breast cancer deaths occurring in developing countries [14]. The overall 5-year survival rate of breast cancer in developed countries exceeds 80%, but decreases in developing countries. For instance, it is claimed to be below 70% in India and less than 50% in South Africa [15]. This highlights that breast cancer outcomes are disproportionately poorer in developing and undeveloped countries compared to developed countries.

The disparity in breast cancer outcomes stems from many factors, including early detection, therapy access, genetics, healthcare availability, insurance, risk factor prevalence, screening rates and care quality [16–18]. Conversely, delayed diagnosis is prevalent in developing countries, with more than 50% of breast tumours diagnosed after advanced stage and metastasis. Various factors lead to delayed diagnosis in these countries, primarily stemming from insufficient cancer literacy among communities and healthcare providers, coupled with complex and fragmented healthcare systems that are difficult to navigate due to financial constraints [19]. Advanced disease at diagnosis leads to reduced survival chances and requires more extensive and expensive treatment, which may be unattainable, hence intensifying the burden on vulnerable healthcare systems [20].

**CRC**: In the United States, CRC is second only to lung cancer in the cause of cancer-related deaths. CRC disparities in the United States are complex and multifactorial, resulting from the interplay of socioeconomic factors, race and ethnicity, lifestyle, access to healthcare and systemic biases within the healthcare system [21]. These factors contribute to significant inequities in CRC incidence, treatment and survival rates, particularly among marginalized communities. In the United States, Blacks have the highest incidence and mortality rates of CRC relative to other racial groups [22]. Comparatively, Blacks have about a 20% higher risk of getting the disease and a 40% higher risk of dying from it than other groups [22]. SES plays a critical role in shaping CRC outcomes. Individuals with lower SES face financial barriers that limit access to preventive care, timely screenings and effective treatments. Poverty often restricts access to nutritious food, safe neighborhoods and health insurance, while limited health literacy reduces awareness of CRC risk factors and the importance of early detection. Communities with fewer healthcare facilities are particularly vulnerable, exacerbating delayed diagnoses and poor outcomes. Consequently, minority underserved populations are usually limited to unhealthy food and reduced access to screening and proper healthcare facilities, predisposing them to CRC and other diseases [23].

With early detection and subsequent intervention, the CRC survival rate can be above 90%. Socioeconomic disparities limit fair access to cancer screening and treatment, disproportionately affecting Blacks, American Indians and other underserved groups. Black [24]. Research indicates that minority underserved populations in the US typically experience delayed diagnoses relative to the general population. A study by [25] Carethers suggests that at the time of diagnosis, 37% of Blacks exhibit localized CRC, which is readily treatable through surgical or endoscopic intervention, in contrast to 38% of Caucasians. Another study by Siegel *et al* [26] shows that about 32% of Blacks have localized disease in comparison to 36% of Caucasians, for whom treatment typically involves a combination of surgery and chemotherapy. Furthermore, 26% of Blacks present with metastatic CRC, compared to only 22% of Caucasians, where the disease often results in mortality [26]. The 5-year survival rate of CRC among Blacks has typically been 6%–12% lower than that of Caucasians, with survival rates of 61% and 67% in 2017, respectively [25]. These statistics demonstrate that disparities in CRC mortality disproportionately affect Black populations. The data further suggest that these differences may be partly explained by later-stage diagnoses and socioeconomic disadvantages within these communities.

Non-modifiable genetic variables can also cause CRC disparity among populations. These genetic factors usually involve a notable ancestry history of cancer. Black and Hispanic Americans with an ancestry history of cancer exhibit the lowest probability of engaging in screening, hence leading to delayed detection of the condition [27]. Moreover, Blacks are less informed about their ancestry cancer history compared to Caucasians [28], and relatives are less inclined to disclose the discovery of colonic polyps [25, 29].

Addressing CRC disparities will require a multifaceted approach focusing on expanding access to affordable screenings and treatment, particularly in underserved areas. Improving healthcare equity through culturally tailored education, outreach and interventions can enhance prevention and early detection efforts. Training healthcare providers to recognize and address implicit biases is crucial for ensuring equitable treatment for all patients. Additionally, community-based initiatives that promote healthy lifestyles, such as increasing access to nutritious food, creating safe spaces for exercise and offering smoking cessation programs, can reduce CRC risk.

**Lung cancer**: Lung cancer is a predominant cause of cancer-related mortality globally, marked by unregulated cellular proliferation in lung tissues [30, 31]. This malignancy is the most prevalent worldwide, with marked incidence and mortality rates, especially among populations with socioeconomic disadvantage [32, 33]. Primarily, lung cancer is classified into non-small cell lung cancer (NSCLC) and small cell lung cancer, with NSCLC being the more common kind [31, 33]. Patients with interstitial lung disease (ILD) exhibit an elevated risk of lung cancer development; however, the pathophysiology linking ILD and lung cancer remains ambiguous [34]. Moreover, a significant correlation exists between lung cancer and chronic obstructive pulmonary disease (COPD); individuals with COPD possess a risk of acquiring lung cancer that is up to five times greater than that of individuals with normal lung function [35]. The primary risk factors for lung cancer include smoking, exposure to environmental carcinogens (such as radon, asbestos and air pollution), genetic factors and occupational hazards [31, 33, 36, 37]. Early detection and proper staging are crucial for effective management, which may involve surgery, chemotherapy, radiotherapy or a combination of these modalities [33, 38]. Prevention strategies include smoking cessation, screening for high-risk individuals and potentially increasing vegetable and fruit intake [31, 37].

Disparities in the development, diagnosis, treatment and outcomes of lung cancer are mainly observed among racial, ethnic and other minority groups, women, HIV-positive individuals and the aged [39]. Blacks are disproportionately impacted, exhibiting elevated incidence and diminished survival rates relative to other racial and ethnic groups [40]. Gender differences in lung cancer have recently become a public health issue. The female-to-male incidence rate ratio has consistently risen from 2001 to 2019, with females aged 0–54 exhibiting higher overall incidence rates than men in recent years across most races and locations [41]. Moreover, geographic variations are evident, with the Southern region of the United States exhibiting more significant incidence rates compared to other regions of the country [42]. The foundation of these disparities is intricate and multifaceted, encompassing social inequities intertwined with genetic and biological factors. Social determinants of health (SDoH), including low SES, absence of health insurance and restricted access to healthcare, disproportionately affect racial, ethnic and rural populations [39, 43]. Environmental variables, including radon, industrial pollutants and air pollution, exacerbate inequities, notably impacting working-class communities and marginalized groups [44]. To address these disparities and improve lung cancer survival rates, a multilevel approach is necessary, focusing on smoking cessation, improved access to high-quality healthcare and culturally specific preventive and treatment strategies [42, 45, 46].

**Prostate cancer**: Prostate cancer is the predominant malignancy in males in industrialized countries and the second foremost cause of cancer-related mortality among American men [47, 48]. Prostate cancer ranks as the second most prevalent cancer among males worldwide, with considerable disparities in incidence across various countries [49, 50]. The risk factors encompass modifiable and non-modifiable components [51, 52]. The risk significantly escalates with age, rising from 0.005% in men under 39% to 13.7% in men aged 60 to 79 [52]. Black men exhibit a mortality risk 2.5 times higher than that of Caucasian men [53]. The disproportionate disparity in mortality rate among Black men is mainly driven by delayed diagnoses, usually at more advanced stages, and are less likely to obtain curative treatments. Prostate cancer exhibits a significant heritable component; however, the etiology is ambiguous in the majority of instances [48]. Identified risk variables encompass age, race/ancestry and family history, with familial clustering noted in 10%–20% of males diagnosed with prostate cancer [48, 54]. Genetic research has discovered more than 110 prevalent risk-associated genetic variations and uncommon mutations that contribute to prostate cancer susceptibility [48].

Interestingly, modifiable factors play a crucial role in prostate cancer development. An exceptionally high intake of dairy products, red meat and saturated fats is associated with increased risk [55–57]. Conversely, consumption of fruits, vegetables (especially tomatoes and cruciferous vegetables) and soy may have protective effects [51, 55]. Environmental factors such as chemical exposure and heavy metals may also contribute to cancer risk [49, 50]. Surprisingly, diabetes mellitus was found to be associated with decreased prostate cancer risk in one study [58]. These observations are suggestive that an interplay of SES and genetic disposition drives an uneven incidence and mortality rate of prostate cancer against the Blacks.

Although socioeconomic position and healthcare access are pivotal in prostate cancer inequalities, recent studies indicate that merely equalising healthcare access may not wholly eradicate racial health disparities [59]. Biological distinctions among ancestral populations, including heightened androgen receptor signaling, genomic instability and inflammatory signaling in Black men, substantially contribute to these discrepancies [59].

A diversified approach is essential to rectify these inequities. This will include enhancing multi-center interdisciplinary research to reduce the disparities created by socioeconomic and biological factors [59], enrolling and retaining a more significant number of Black men in prostate cancer clinical trials and observational studies [60], and formulating personalised medicine strategies that target genomics and epigenetics [61]. Comprehending the factors contributing to elevated prostate cancer mortality in Black males continues to be a significant obstacle in tackling disparities in outcomes of prostate cancer [62].

**Cervical cancer**: Although cervical cancer is very preventable, it still ranks fourth among the most prevalent cancers in women globally, with approximately 660,000 diagnoses and 350,000 deaths in 2022 [63]. The human papillomavirus (HPV), a sexually transmitted virus transferred via skin-to-skin and genital contact, is thought to account for over 90% of cervical cancer cases. Most infections in young adults are asymptomatic and masked by the immune system, leading to negative test results within 1 to 3 years after initial HPV infection. Long-lasting infections that endure for 5 years or longer may result in the formation of precancerous lesions, potentially leading to cancer development [64]. Interestingly, while cervical cancer is often associated with older women, studies have shown that a significant number of cases occur in younger women. For example, in one study, 17.6% of patients with adenocarcinoma and adenosquamous carcinoma were younger than 50 [65]. Risk factors for cervical cancer include HPV infection, poverty, lower education levels, multiparity, tobacco use, malnutrition and poor genital hygiene [66]. Additionally, a study using machine learning techniques identified age, number of sexual partners and hormonal contraceptives as having a more significant influence on cervical cancer diagnosis compared to other risk factors [67]. Prophylactic HPV vaccine and cervical cancer screening are the established standards for disease prevention and early diagnosis [64].

In the United States, notable racial and ethnic disparities are present in cervical cancer outcomes and treatment. Non-Hispanic Black and Hispanic women exhibit elevated incidence and mortality rates relative to non-Hispanic White women [68]. The major driver of the observed disparity in the United States is socioeconomic factors built into the delayed treatment of underserved populations. Minority groups such as Black and Hispanic women are less likely to receive guideline-based treatment and face longer wait times for definitive treatment [69]. Data shows that Black patients experience significantly longer Time to Treatment Initiation (TTI) (33.5 days) compared to Caucasian patients (30.4 days). Hispanic ethnicity is also associated with increased treatment delays, with Hispanic patients experiencing longer TTI (34.8 days) compared to non-Hispanic patients (30.6 days). These disparities appear to be part of broader socioeconomic barriers to care, as factors such as Medicaid coverage, lower education levels and distance from treatment facilities also contribute to increased TTI. The increasing TTI trend affects patients across all disease stages and facility types, suggesting a systemic issue in cancer care delivery that disproportionately impacts minority populations [70].

Overall, the patterns of disparity observed across various cancer types are remarkably similar, largely driven by the complex interaction of several factors. These disparities often reflect deep-rooted systemic barriers that, over time, contribute to persistent disadvantages for underserved populations.

## Factors affecting cancer disparities

### Socioeconomic factors

Among the many factors leading to cancer disparities, social and economic factors are among the most critical. The socioeconomic gradient in cancer disparities exhibits complex patterns that vary between and within different territories [71]. In general, the incidence rate of all cancers increases with the increasing levels of national socio-economic development; that is, undeveloped countries are more likely to record a decrease in cancer incidence rates compared to developed countries. However, this gradient is not well defined for survival, as mortality rates are usually characterised by substantial heterogeneity and several exceptions. For most cancers, the mortality rate is disproportionately higher in countries with lower SES. Even within the same country, the mortality rate is usually higher among groups with low SES [71]. A solitary component cannot adequately encapsulate the intricate characteristics of SES; thus, educational attainment, occupational standing and income level are interrelated in delineating an individual’s SES [72]. As a result, numerous epidemiological studies evaluate a mixture of these factors when assessing their influence on cancer risk.

Although there are inconsistencies between educational attainment and income, individuals with elevated literacy levels predominantly occupy managerial and high-level professional positions associated with higher salaries [73]. Income level is a significant determinant of health disparities, and in addition to reflecting the work category/position, it also predetermines the affordability of healthcare. Hence, poverty status is the most significant social determinant of health, leading to disparities in cancer incidence, prevalence and mortality through various mechanisms, including access to and affordability of quality healthcare, early cancer diagnosis and associations with tobacco and other substances [74]. Key factors leading to elevated mortality rates in low-income demographics captured under healthcare costs and marginalisation include inadequate access to housing, lack of insurance, low financial resources and credit for out-of-pocket expenditures and essential services. Furthermore, the absence of screening for at-risk populations is associated with prevailing attitudes, beliefs and fears [75]. The prevalence of specific cancer forms also varies with SES. Infection-related cancers are typically more prevalent in populations of low SES and residents of low-income countries. In contrast, cancers such as those of the breast, prostate, thyroid, colon and rectum are historically more prevalent in HICs. [71].

The study by O’Connor *et al* [76], which analysed a sample of 3,135 counties across several US states, found that median salaries varied from $22,126 to $121,250 annually. In comparison to counties in the high-income category (median income, $55,780), those in the low-income category (median income, $33,445) exhibited more significant numbers of residents who were non-Hispanic black, resided in rural areas or had a history of poverty and/or illness. Their study indicates that cancer mortality rates in the US vary considerably among counties according to their average income level. The average cancer mortality rate was 185.9 per 100,000 individuals in HICs, in contrast to 204.9 and 229.7 per 100,000 individuals in medium- and low-income counties, respectively. In a different study by Wang *et al* [77], most cancer prevalence, including breast and lung cancers, are found to predominate among high and middle-income earners compared to low-income earners. These observations suggest that higher prevalence in some cancers among higher income groups may be partly due to better access to screening and detection rather than actual disease occurrence. The statistics from the two studies indicates that even though the mortality rate of cancer is inversely proportional to average income, the effect of on cancer prevalence seems to be directly proportional.

### Genetic factors

Generally, sex and gender are modifiers of health outcomes and contribute to disparities in disease development and progression [78]. The most significant and most apparent cancer disparities are seen in sex/reproductive-related cancers such as breast cancer, prostate cancer, ovarian cancer and so on. These classes of cancer are almost exclusively attributed to a unique gender due to the associated reproductive organs affected [79, 80]. Generally, males have a higher susceptibility to cancer than females, resulting in a global male preponderance of 2:1 for many non-reproductive cancers, underscoring the significant cancer imbalance skewed towards males [79]. Sex hormone signaling and oncogenes encoded by the Y chromosome are determinants of cancer disparities related to sex and gender; therefore, sexual dimorphisms in cancer genetics have been identified across several cancers [81]. The androgen receptor plays a crucial role in the advancement of liver illnesses such as fatty liver, cirrhosis and liver cancer, aligning with a global male predominance in liver cancer incidence ranging from 2:1 to 7:1 [82]. In addition, the efficacy of cancer treatment may vary between females and males, as the therapeutic advantage of immune checkpoint inhibitors in many cases is contingent upon sex, with these treatments yielding more significant benefits for men [83]

Although gender is an established determinant of cancer disparities, the biological mechanisms underlying sex-related cancer disparities are still an area of research interest. Sex and gender are therefore well considered during diagnostic, preventive and therapeutic strategies for diseases [78]. Cancer health disparities are also affected by sexual behaviour. For instance, anal cancer incidence rates are rising in both men and women worldwide, necessitating population-based preventative strategies, including the promotion of safe sexual practices and HPV vaccination [84].

In addition to gender and sex factors in cancer disparities, other genetic elements such as genetic ancestry, hereditary lineage and natural selection contribute to population variations in immune responses to pathogens, thereby significantly influencing disparities in cancer incidence and mortality [85, 86]. For instance, one of the distinguishing factors between Black and Caucasian cancer patients is their pan-cancer mitochondrial activity, indicating a potential ancestral connection [87]. Furthermore, studies on human populations have demonstrated the correlation between ancestral lineage and the expression levels of specific inflammatory cytokines, which may be significant in the development and progression of cancer, thereby potentially contributing to disparities in cancer outcomes [88, 89]. A gene expression study conducted by Storey *et al* [90] on lymphoblastoid cell lines from individuals of European and West African ancestry revealed that these polymorphisms can aggregate in cancer-related pathways. Consequently, genetic variations among communities may result in population-specific vulnerabilities to prevalent diseases, such as certain cancers, due to their influence on the transcriptome [91].

Almost every class of cancer has been documented to exhibit some level of genetic disparities, either because they directly affect reproductive organs, are impacted by sex chromosomes or due to clusters of gene differences that are peculiar to specific ancestries. However, studies have shown that some cancers are more likely to be impacted by genetic disparities. Breast, colorectal, liver, prostate, kidney, lung, cervical and pancreatic cancers are among the most documented cancers for genetic disparities [12, 92].

### Geographic factors

Geographic factors significantly influence cancer incidence, prevalence and outcomes, driven by environmental, lifestyle, cultural factors, demographics, genetic predispositions, infectious agents and socioeconomic and healthcare-related factors. HICs benefit significantly from routine cancer screening programs, contributing to lower mortality rates. In contrast, lower-income countries (LICs) face substantially higher mortality because of notable barriers that delay diagnoses and limit treatment accessibility. This inequality is entrenched in systemic challenges that impact the entire spectrum of cancer care, from prevention to treatment. For instance, HPV has been observed to elevate cervical cancer rates in some countries of Asia and Africa, attributable to inadequate vaccination initiatives and insufficient screening and public awareness efforts [93]. Breast cancer mortality rate for women in low-income countries is 17% higher than those in industrialized countries (15.0 against 12.8 per 100,000, respectively), with the highest rates observed in Melanesia, Western Africa, Micronesia/Polynesia and the Caribbean. Significant increases are observed in sub-Saharan Africa, accompanied by escalating mortality rates [15]. The statistics indicate that cancer mortality rates are likely to be higher in regions characterized by lower income and reduced SES. This breast cancer mortality is notably higher in LICs and rural areas due to limited access to breast cancer screening, advanced treatments and public awareness programs [94].

The impact of geography on cancer outcomes is well demonstrated in a study examining the survival differences of NSCLC patients from different geographic areas by [95]. Findings indicate that patients in rural and small-town areas with surgically treated NSCLC have lower survival results compared to their urban and metropolitan counterparts, possibly attributable to a higher frequency of smoking [95]. These disparities in lung cancer outcomes between rural and urban populations, therefore, present a public health concern and reflect differences in risk factor prevalence, healthcare access and treatment availability. However, some studies found no survival difference for patients with advanced-stage lung cancer, suggesting the disparities may be more pronounced for early-stage disease [96]. The findings highlight the need for targeted smoking cessation programs and improved access to surgical treatments in rural areas to reduce lung cancer mortality and address healthcare disparities between rural and urban populations. In a different study by Loehrer *et al* [97] on the availability and accessibility of healthcare facilities in metropolitan and nonmetropolitan areas, metropolitan beneficiaries exhibited a higher rate of cancer-directed surgery (22.1%) compared to their nonmetropolitan counterparts, including micropolitan (18.7%), small town (17.5%) and isolated rural (17.8%) residents (*p* < 0.001). These numbers suggest that residing in rural areas is consistently associated with reduced access to essential medical facilities, which in turn increases the risk of cancer mortality. The disparities in cancer-directed surgery rates and outcomes between metropolitan and nonmetropolitan patients with lung cancer highlight significant healthcare access issues in the non-metropolitan areas. Additionally, patients in nonmetropolitan areas experience a more extended time from diagnosis to surgery and lower odds of receiving minimally invasive surgery. Furthermore, nonmetropolitan residents correlated with increased postoperative emergency department visits, indicating potential complications or inadequate follow-up care [97].

For certain types of cancers, like CRC, geography has been noted to affect the onset, probably due to the impact of urbanisation on diet and lifestyles. Generally, cancers that are heavily impacted by diets, such as colorectal, liver, lung, pancreatic, stomach, oesophageal and nasopharyngeal cancers, are more likely to have a higher early onset in urban and civilized regions compared to rural and undeveloped areas. Race/ethnicity and region in the United States are major drivers of disparities in early-onset CRC. Blacks, Hispanics/Latinos and residents of certain areas in the Southern U.S. experience a disproportionate incidence of CRC diagnosed at younger ages [98]. On a global scale, early-onset CRC incidence has steadily increased among individuals younger than 50 years in developed and more civilized countries, including the United States and other HICs. Data on the annual percent increase in the early onset of CRC in the US (2.2%), New Zealand (4.0%), Canada (2.8) and Australia (2.8) compared to the global average of 1% indicates the high rising early onset of CRC in geographies with heavy civilisations [99, 100]. This can be attributed to adopting Westernized diets, exposure to more pollutants and lifestyles associated with increased civilisation.

Furthermore, studies have shown that geography can combine with other factors to drive cancer disparities. For instance, developed countries generally have higher cancer incidence rates than developing countries, suggesting the influence of lifestyle and environmental factors within such geography [101]. Within the United States, spatial analyses have revealed geographic patterns of cancer risk [102], and studies have shown that racial and ethnic clusters influence these geographic patterns in determining disparities in certain cancers known to persist across different geographic regions. Black men, for instance, experience both higher incidence rates and more aggressive forms of prostate cancer compared to other racial groups [101]. This disparity is most evident in the United States, where Black men face over two-fold heightened risk of mortality from prostate cancer relative to all other racial groups [59]. Environmental factors, including climate, may contribute to these geographic disparities.

Additionally, SES, healthcare access and cultural factors vary across geographic regions, further contributing to the observed disparities in cancer outcomes [59]. These inequalities highlight an urgent need for interventions to improve screening, diagnosis and treatment access in the disproportionated regions. Geographic access to healthcare significantly affects health outcomes; urban areas typically have more concentrated healthcare resources and offer better access to diagnostic and treatment services. Conversely, rural areas face substantial barriers, including fewer healthcare providers, longer travel distances and limited infrastructure, contributing to lower treatment and survival rates [103]. Healthcare disparities further exacerbate these regional inequities. Restricted access to quality diagnostic tools and treatments is a significant challenge in economically disadvantaged regions. This disparity frequently leads to delayed diagnosis, more advanced stages of disease at presentation and worse outcomes. Addressing these disparities requires a multi-faceted approach, including expanding healthcare infrastructure, promoting early detection programs and ensuring equitable access to quality care, particularly in low-income and rural regions.

### Behavioural and lifestyle factors

Behavioural and lifestyle variables substantially affect the risk of multiple cancers, including breast, cervical, prostate, lung and CRCs. Several studies have uncovered the intricate interrelations and cumulative impacts of factors such as smoking, food, physical activity, alcohol intake and substance misuse on cancer incidence and mortality. Comprehending these linkages is essential for efficacious preventative methods.

Smoking is associated with a heightened risk of multiple cancers, including breast, lung, colorectal and brain cancers. The interplay of smoking with obesity or alcohol intake raises cancer risk, especially in men [104]. Research demonstrates that the most significant rates of Disability-Adjusted Life-Years associated with smoking were recorded in Bulgaria and Monaco [105]. The cessation of smoking is correlated with enhanced survival rates in lung cancer patients [106]. Cigarette smoking is linked to an elevated risk and diminished prognosis for CRC, especially in Blacks [107].

Smoking is also linked to a higher risk of brain tumours [108] and may influence breast cancer risk through its effects on mammographic density [109]. In addition to smoking, other substance abuse, including alcohol and illegal drugs, is prevalent among university students and associated with various health and behavioural issues [110]. The combined effects of alcohol consumption and cigarette smoking accounted for 20% of rectal cancer cases in an Italian study [111]. Alcohol consumption is a well-established risk factor, with significant associations found for both premenopausal and postmenopausal breast cancer [112]. Substance abuse exacerbates the risk of these cancers and can negatively impact treatment outcomes [112]. In rectal cancer, taking more than three bottles of alcohol per day increased risk by 74% overall and 180% in females [110, 111]. This trend suggests that alcohol consumption and other substances that deplete the body’s antioxidant defenses may contribute to increased incidence and mortality across multiple cancer types.

A healthy diet and regular physical activity are associated with reduced cancer risk. A balanced diet with sufficient macro and micronutrients is essential for optimal immune function, while high-energy ‘Western’ diets and obesity increase the risk of cancers and potentially other diseases [113]. For instance, obesity linked to poor dietary choices increases the likelihood of developing breast and CRCs [114]. Dietary patterns characterized by elevated red meat consumption and diminished fruit and vegetable intake are associated with heightened risks of colon, bladder, breast and kidney cancers [106]. Physical inactivity has been demonstrated to increase cancer risk, highlighting the necessity for lifestyle changes [115]. In CRC, a higher body mass index (BMI) correlated with a reduced mortality risk in rectal cancer patients; also, a lower BMI was associated with an elevated mortality risk in colon cancer patients [116]. Regular physical exercise has been demonstrated to lessen the incidence of breast cancer by approximately 30%–40% [112].

These behavioural and lifestyle factors are modifiable risk factors that can be targeted for cancer prevention and improved prognosis. Public health strategies should focus on reducing smoking rates, limiting alcohol consumption, promoting balanced diets, encouraging physical activity and addressing substance abuse to reduce cancer risk and improve outcomes across various cancer types [113, 117, 118].

### Environmental and occupational factors

Environmental and occupational exposures significantly contribute to cancer disparities among different populations. These disparities are shaped by systemic inequities in environmental policy, workplace protections and access to healthcare. Disadvantaged communities often bear the brunt of harmful exposures due to socioeconomic and racial inequalities, leading to disproportionate cancer burdens [119].

Environmental factors like air pollution, industrial waste and proximity to hazardous sites contribute to cancer risk. Marginalized populations are more likely to reside near industrial facilities or highways, increasing their exposure to carcinogens like benzene, asbestos and polycyclic aromatic hydrocarbons (PAHs). For example, studies show that Black and Hispanic communities experience higher levels of air pollution compared to White populations, correlating with increased lung cancer risk [120].

Additionally, agricultural workers, who are often from immigrant or low-income groups, face significant exposure to pesticides and herbicides. Chronic exposure to these chemicals is associated with cancers such as non-Hodgkin lymphoma and leukaemia [121]. The environmental impact on cancer disparities strongly underlines the importance of socioeconomic inequalities in cancer outcomes. This impact is exacerbated by a lack of adequate safety regulations and enforcement by local and national authorities. The environmental impact on cancer burden is mainly through socioeconomic and societal factors, which we can summarize into three categories: occupational exposures, systemic inequalities and policy implications and living conditions, pollution and carcinogens.

**Occupational exposures:** Workplace exposures can contribute to cancer disparities through jobs with high cancer-related risks, such as construction, manufacturing and mining, which lower-income individuals and racial minorities disproportionately occupy. Workers in these industries are often exposed to silica, asbestos and diesel exhaust carcinogens. For example, Hispanic workers in the U.S. construction industry have higher rates of occupational lung cancer due to inadequate protections and limited access to healthcare [1]. Women in low-wage jobs, such as hairdressers and cleaners, frequently encounter harmful chemicals that increase their risks for breast and ovarian cancers. These risks are amplified by insufficient worker education and lack of access to protective equipment [122].

**Systemic inequities and policy implications:** Cancer disparities linked to environmental and occupational exposures reflect broader systemic inequalities. Communities of color and low-income populations often have limited access to preventive measures, early detection and high-quality care. Policies that fail to prioritize environmental justice perpetuate these inequities. For example, the delayed cleanup of contaminated sites under the United States Environmental Protection Agency Superfund program of the 2022 Fiscal year disproportionately affected minority communities [123].

**Living conditions, pollution and carcinogens**: Air, water and soil pollution, which are significant sources of exposure to carcinogens, disproportionately affects low-income and minority populations. Proximity to industrial facilities, landfills and highways increases exposure to substances such as benzene, asbestos and PAHs, which are associated with cancers of the lung, liver and bladder [124]. Urban areas with higher concentrations of minority populations experience elevated levels of fine particulate matter and nitrogen dioxide, contributing to increased risks of lung cancer [122]. Blacks are 1.5 times more inclined to reside in regions with substandard air quality than Caucasians [119]. Contaminated water sources, such as those with high arsenic or industrial runoff levels, are linked to skin, bladder and kidney cancers. For example, in Flint, Michigan’s water crisis, which disproportionately affected Black residents, highlights systemic environmental injustices [125]. Poorer neighborhoods often have less greenery and more heat-retaining infrastructure, amplifying heat stress and air pollution. These factors indirectly contribute to cancer risks by increasing oxidative stress and inflammation [126]. Policies to improve housing conditions and reduce environmental burdens in vulnerable areas are crucial to mitigating disparities.

Overall, this section clearly shows that cancer disparities are largely driven by an intricate interplay of genetic, socioeconomic, geographic, behavioural and environmental factors, with systemic inequalities amplifying these differences, particularly among underserved populations. We emphasize that these disparities are entrenched in systemic structures that, over time, manifest as barriers to access and quality of care, thereby increasing mortality risk.

## Advocacy and interventions

Addressing cancer disparities requires a multifaceted approach involving current policies, advocacy efforts and community programs. While significant progress has been made in reducing cancer mortality, cancer disparities have persisted, necessitating targeted policy changes and community engagement to ensure equitable access to care and resources. The U.S. has implemented policies to reduce health disparities, particularly in cancer care, but inconsistent application has led to varied outcomes [127, 128]. Policies must address SDoH and ensure access to screening and treatment, as disparities contribute to significant mortality rates and economic costs [130]. Community-based organisations, such as cancer councils, play a crucial role in policy development and resource mobilisation, enhancing community engagement in health initiatives [129]. Programs like community health fairs and outreach initiatives have effectively identified barriers and improved access to evidence-based cancer care [130]. Policies should prioritize funding for local initiatives, enhance community engagement in policy making and ensure that interventions are tailored to the needs of underserved populations [131]. Focusing on community-academic partnerships can strengthen advocacy efforts and promote sustainable policy changes to eliminate disparities [131].

Because cancer disparities among racial, ethnic and socioeconomic groups have become a pressing public health issue in recent years, it has become expedient that more public health policies and advocates are in place to help bridge the gap in cancer burden among different groups and regions. We have outlined several drivers of these disparities, including factors such as access to care, genetic predispositions, cultural differences and SDoH. In the past, intervention programs have built strategies around the determinants of health inequalities to achieve successful interventions. So, we critically look into some community-based interventions to outline the approaches for successful interventions in the past and highlight the overwhelming need for more interventions. Community-based programs have been instrumental in mitigating these disparities by tailoring interventions to the needs and preferences of underserved populations. Community-based interventions are usually built to target structural inequalities in health. Other studies and our current review have shown that cancer health disparities are deeply rooted in structural inequities. In addition, marginalized communities are historically known to face several structural inequalities, such as limited access to healthcare services, mistrust in the healthcare system, socioeconomic and cultural barriers.

### Successful community-based programs

**Patient navigation programs (PNPs)**: PNPs aim to help individuals overcome barriers to accessing cancer screening by providing support and guidance throughout the process, including scheduling appointments, addressing transportation issues, explaining procedures and navigating insurance complexities, ultimately increasing screening rates, particularly among populations with historically lower screening rates due to socioeconomic factors or cultural barriers; essentially acting as a dedicated point of contact to guide patients through the healthcare system to ensure timely screening and treatment [132].

The PNP is an evidence-based intervention designed to assist patients and their support systems in navigating health systems, health information, appointments, transportation, health finances and other barriers to care [132]. PNPs have consistently and effectively addressed cancer inequalities among diverse communities. A case study on CRC indicated that PNPs mitigated cancer disparities and diminished the overall CRC burden within the study group (Table 1) [134]. A meta-analysis conducted by Nelson and associates evaluated 28 US trials that assessed the efficacy of PNP in enhancing CRC screening rates relative to standard care or alternative control groups [134]. The studies, summarized in Table 1, indicated an elevated screening rate in patients receiving PNP compared to those without, with a risk ratio of 1.64 and a 95% confidence interval of 1.42–1.92 [134]. Increasing awareness and access to cancer screening is one of the significant goals and mechanisms of implementing the PNP. To describe a case study that demonstrated the effectiveness of this intervention method, Patient Navigation Services to Increase Breast, Cervical and CRC Screenings and Advance Health Equity, reported by CPSTF [132], used a systematic review of 34 studies between 2016 and 2021 to describe the effectiveness of PNPs.

PNPs are developed to reduce barriers to care. PNPs use trained clinicians to assist patients in accessing screening, treatment and follow-up care. For example, the Harlem Cancer Prevention Program successfully increased breast and cervical cancer screening rates among Black women by offering patient navigation services [135]. A similar model has been applied in Hispanic communities to address language barriers and improve CRC screening uptake [136].

**Community-based education and outreach**: Community-based education and outreach are essential for mitigating cancer disparities among populations. It functions by actively involving underserved populations to deliver culturally pertinent information regarding cancer prevention, screening and treatment and tackling the SDoH that frequently lead to unequal cancer burdens within communities.

Mechanistically, it entails actively engaging with populations impacted by cancer inequalities across all program sectors, including research, intervention creation, implementation and assessment. It embodies the importance of the community members in providing information, experiences and viewpoints that may inform and direct initiatives to address inequities. The interaction became an active cooperation with the members rather than a mere passive recipient paradigm, enabling communities to become part of decision-making in health-related processes. In providing interventions, community participation is vital for its effectiveness in guaranteeing that interventions and tactics are culturally suitable and sensitive to the distinct needs of varied communities. This is achieved through actively involving the members to cultivate trust, advance health literacy and improve the acceptability and durability of interventions through the active involvement of community people. In addition, it enables the identification of community-specific obstacles, advantages and resources, resulting in more effective and fair strategies for mitigating cancer inequalities.

**Cancer education program focused on culture and ethnicity, a case study [137]:** This case study analyses a community engagement campaign that created culturally and ethnically adapted cancer education programs for community members. The study emphasizes cultural values, including religious and linguistic preferences, in communicating effectively and implementing community-based intervention techniques to reduce cancer inequalities. The effort sought to tailor instructional materials and messages to the distinct cultural environment of the target populations [137]. The research emphasized the deficiency of cancer awareness within Indian populations, particularly regarding curability, preventability and accessible screening techniques. In addition, the study highlighted the insufficient understanding of risk factors associated with most cancers linked to tobacco and alcohol consumption. The study indicated a favorable disposition towards screening methods throughout the Indian populace. Nonetheless, despite this favorable disposition, the actual screening processes were shown to be inadequate. However, the research recognized the potential advantage of fostering community-level awareness to improve screening.

The case study demonstrated the significance of education and residential location (rural or urban) in shaping cancer outcomes. It uses study materials that were meticulously crafted to be culture-conscious and linguistically accessible, including recognisable signs, customs and practical instances that are in line with the culture and tradition of the community [138]. The principal target of the culturally adapted approach was to close the gap in knowledge and encourage routine cancer screening. The research indicated that culturally customized cancer education initiatives positively enhanced their inclination to preventive measures, routine screening and early detection among the targeted communities. The case study revealed that community participants became more confident in undertaking proactive measures for cancer prevention and participating in regular checkups. This project gave the community members a sense of belonging as their cultural values and viewpoints were integrated into the instructional materials [139].

In summary, advocacy and intervention efforts targeting cancer disparities have focused on increasing awareness, improving access to screening and treatment, and addressing SDoH through community-engaged strategies. Successful programs often incorporate patient navigation, culturally tailored education and policy initiatives designed to overcome barriers faced by underserved populations. Despite progress, sustained multi-sectoral collaboration and systemic changes are essential to reduce cancer disparities and improve equity in cancer outcomes globally.

## Limitations and recommendations

Although our narrative review provides a comprehensive synthesis of disparities across major cancers, it is not exhaustive. Our literature search was limited to published and accessible data sources, which may not fully capture emerging findings or unpublished regional studies. Furthermore, this review emphasizes adult cancers, specifically breast, colorectal, lung, prostate and cervical cancers, while paediatric cancers and rare malignancies were not covered. As such, some relevant contexts of cancer disparities, particularly those affecting younger populations or less common cancer types, remain beyond the scope of this analysis. Nonetheless, the insights presented here offer valuable foundations for future research that broadens coverage across age groups and cancer types. Further studies should expand to include underrepresented cancer types, such as paediatric and rare malignancies, to provide a broader and more inclusive understanding of global cancer inequities. Increased investment in longitudinal and multi-center studies across diverse populations is also essential for capturing temporal and geographic variations in disparity patterns.

## Conclusion

There are various statistical data to support the existence of cancer disparities and its status as a public health concern around the globe. Our study reveals that disparities in cancer incidence and outcomes are consistent across major types of cancer and are mainly driven by an interplay of biological and SDoH. The impact of cancer disparities is heavy on underserved groups that are usually marginalized and are unable to fully access the social and economic mainstream of society. In addition to affecting underserved groups, cancer disparities also have a spillover effect that can impact the overall health status of society. Combating cancer disparities will, therefore, not only improve the health status of the underserved groups but will improve the overall health status of the society by improving health-related quality of life and decreasing medical costs. Eliminating disparities demands major changes in health advocacy and interventions. This study is indicative that epidemiological data on disparities can inform the craft of practical strategies that improves cancer statistics among underserved populations. This approach requires coordinated efforts across research, policy, healthcare delivery and community engagement.

## Conflicts of interest

**The authors declare no conflicts of interest.**

## Funding

**This research received no external funding.**

## Figures and Tables

**Table 1.** Summary of outcomes from community-based patient navigation and education interventions on breast, cervical and CRC screening rates among underserved populations [132, 134].

Cancer type	Data analysis	Number of studies	Tests	Results	Inference
Breast	MA	10	-	1.32 (RR) 1.08–1.62 (95% CL)	Positive
	MA and IQI	11	-	AD: 12.0 pct points IQI: 9.7 to 24.2 pct points RD: 54.5% IQI: 14.5%–75.3%	Positive
Cervical	Median and range	3	-	AD: 22.5 pct pts. RD: 64.5% Range: 9.9% to 67.6%	Positive

Colorectal	MA	26	Any test	RR: 1.82 95% Cl: 1.50 to 2.21	Positive
	Median and IOI	26	Any test	AD: 13.6 pct pts IQI: 7.9 TO 31.8 pct pts RD: 76.2%	Positive
Median and IQI	12	Colonoscopy	AD: 13.9 pct pts IQI: 9.5 TO 26.1 PCT PTS RD: 109.9% IQI: 34.6%–296.2%	Positive
Meta-analysis	Meta-analysis	IT	RR: 1.65 95% Cl: 1.38–1.99	Positive
Median and IQI	12	FOBT or FIT	AD: 12.4 pct points IQI: 4.9 to 18.8 pct points RD: 57.3% IQI: 37.5%–126.8%	Positive
